# Characteristics and outcomes of recurrent atrial fibrillation after prior failed pulmonary vein isolation

**DOI:** 10.1007/s10840-022-01160-w

**Published:** 2022-02-16

**Authors:** Sai Vanam, Douglas Darden, Muhammad Bilal Munir, Omar Aldaas, Jonathan C. Hsu, Frederick T. Han, Kurt S. Hoffmayer, Farshad Raissi, Ulrika Birgersdotter-Green, Gregory K. Feld, David E. Krummen, Gordon Ho

**Affiliations:** 1 Department of Medicine, Division of Cardiology, Section of Cardiac Electrophysiology, University of California San Diego, La Jolla, CA USA; 2 Veterans Affairs San Diego Medical Center, San Diego, CA USA

**Keywords:** Atrial fibrillation, Atrial tachycardia, Catheter ablation, Mapping, Pulmonary vein isolation

## Abstract

**Background:**

The mechanisms for atrial fibrillation (AF) recurrence after pulmonary vein isolation (PVI) catheter ablation are unclear. Non-PV organized atrial arrhythmias (PAC, AT, macro-reentrant AFL) are possible contributors; however the prevalence and effect of their ablation on recurrent AF are unknown. We hypothesize that the identification and ablation of non-PV organized atrial arrhythmias were associated with less AF recurrence.

**Methods:**

Patients who underwent repeat ablation for recurrent AF after prior PVI were retrospectively enrolled. The prevalence and characteristics of PV reconnections and non-PV organized atrial arrhythmias were identified. The outcomes of time to clinical AF recurrence, heart failure (HF) hospitalization, and mortality were analyzed in patients using multivariable adjusted Cox regression.

**Results:**

In 74 patients with recurrent AF (age 66 ± 9 years, left atrial volume index 38 ± 10 ml/m^2^, 59% persistent AF), PV reconnections were found in 46 patients (61%), macro-reentrant atrial flutter in 27 patients (36%), and focal tachycardia in 12 patients (16%). Mapping and ablation of non-PV organized atrial arrhythmias were associated with a reduced recurrence of late clinical AF (adjusted HR 0.26, CI 0.08–0.85, *p* = 0.03) and the composite outcome of recurrence of late AF, HF hospitalization, and mortality (adjusted HR 0.38, CI 0.17–0.85, *p* = 0.02), with median follow-up of 1.6 (IQR 0.7–6.3) years. The presence of PV reconnections or empiric linear ablation was not associated with reduction in clinical AF or composite endpoints.

**Conclusion:**

The ablation of non-PV organized atrial arrhythmias resulted in a reduction of late clinical AF recurrence and composite outcome. In this challenging population, alternate mechanisms beyond PV reconnections need to be considered. Prospective studies are needed.

**Supplementary Information:**

The online version contains supplementary material available at 10.1007/s10840-022-01160-w.

## Introduction

Atrial fibrillation (AF) is the most common arrhythmia in the world and a leading cause of hospitalization and morbidity^1^. Although catheter ablation has been proven to be effective at eliminating AF, recurrences of AF after catheter ablation are common and estimated to be between 15 and 50% after 5 years.^2^ The mechanisms of recurrent atrial arrhythmias after catheter ablation of AF are not well defined. Prior studies have reported pulmonary vein (PV) reconnections as the predominant mechanism of AF recurrence in up to 97% of patients.^3,4^ Consequently, the current standard of care and only Class 1 guideline recommendation during repeat catheter ablation of AF is to assess for PV reconnections.^2^ As technology has improved through the years, including the use of contact force catheters, PV isolation (PVI) has become more durable and effective. Indeed, recent studies have indicated that the incidence of PV reconnections in recurrent AF is not as common as previously cited, occurring in as low as 38% of patients with recurrent AF.^5^ Still, despite durable PV isolation, AF recurrence is still common.^6^

If no PV reconnections are found, there remains uncertainty as to the best strategy to guide repeat ablation. Different techniques have been proposed in this setting, such as empiric linear ablation with a roof line or mitral isthmus line, posterior box isolation, left atrial appendage isolation, targeting ablation of complex fractionated electrograms (CFAE), ablation of provoked non-PV organized atrial arrhythmias commonly associated with non-PVs organized atrial arrhythmias, such as the superior vena cava.^2,4^ It is unclear whether additional ablations beyond PVI have any benefit in outcomes, although guidelines state that operators should consider more extensive ablation in recurrent AF.^7,8^

The aim of the present study is to determine the prevalence of PV reconnections and non-PV organized atrial arrhythmias (atrial tachycardia [AT]/premature atrial contractions [PAC] and macro-reentrant atrial flutter [AFL]) in patients with recurrent AF undergoing repeat AF ablation and evaluate the impact of their ablation on clinical outcomes.

## Methods

### Patient selection

A total of 74 patients who underwent repeat catheter ablation for recurrent AF after initial PVI were analyzed retrospectively at 2 medical centers (University of California San Diego and Veterans Affairs Medical Center San Diego). The inclusion criteria included consecutive patients over 18 years of age who had previously undergone PVI for AF and who developed clinically significant recurrent AF as documented on ECG, telemetry event monitor, and implantable device interrogation. Patients were excluded if they were found to only have atypical AFL without clinical AF. This study was performed in accordance with approved retrospective Institutional Review Board protocols at each institution.

### Clinical mapping and ablation procedure

The prevalence of PV reconnections, atypical AFL, AT, and PACs were identified using standard mapping techniques. Informed consent was obtained prior to all ablation procedures. Intravenous heparin was used to target an activated clotting time of 350–400 s. Electroanatomic mapping systems were used in all cases (CARTO, Biosense-Webster Inc, Diamond Bar, CA; or Ensite™, Abbott Laboratories, Chicago, IL). Esophageal position and temperature were monitored during all left atrial ablations using a multipolar temperature probe (Circa S-Cath, Circa Scientific, Inc., Englewood, CO) positioned in the esophagus behind the left atrium at the level of the ablation catheter, in order to avoid any temperature rise above 38 °C. Left atrial access with an ablation catheter and multi-electrode catheter (Lasso or Pentaray, Biosense-Webster Inc, Diamond Bar, CA or HD Grid, Abbott Laboratories, Chicago, IL) was obtained with single or double trans-septal puncture, performed under direct visualization with intracardiac echocardiography guidance and fluoroscopy.

If recovery of conduction from PVs was observed, repeat PVI was performed using a segmental, circumferential, or both approaches. Next, organized atrial arrhythmias such as AFL, AT, ectopic PAC, or supraventricular tachycardias were mapped and ablated using entrainment mapping techniques and standard activation mapping with a multipolar electrode catheter. High-dose isoproterenol infusion (20 mcg/min) was infused to induce non-PV organized atrial arrhythmias, as previously described.^9,10^ Of note, non-PV organized atrial arrhythmia sources were defined as any focal ectopic beat or tachycardia induced in the atria, regardless whether they directly induced AF.

Additional lesion sets outside the previously ablated regions were performed at the discretion of the operator, including left atrial roof line, mitral valve isthmus line, coronary sinus ablation, and CFAE ablation. Closed and open irrigated and non-contact and contact force sensing catheters were also used at the discretion of the operator. The endpoint of PVI was elimination of all PV potentials and demonstration entrance and exit block by pacing after a 30-min waiting period and elimination of a trigger or a line of bidirectional conduction block if adjunctive ablations were performed.

### Clinical outcomes and follow-up

Data regarding long-term clinical follow-up were obtained through chart review. Recurrence of atrial tachyarrhythmia was defined as AF, AFL, or AT > 30 s occurring > 3 months after repeat procedure lasting for > 30 s on 14–30 day ambulatory monitors at least 3 months, 6 months, and every year after ablation. Anti-arrhythmic medications were continued during the blanking period for 3 months and then discontinued at the discretion of the treating electrophysiologist. Data on heart failure hospitalization and mortality after ablation was collected.

### Statistical analysis

The baseline characteristics of those with any inducible atrial arrhythmia versus no inducible atrial arrhythmia were reported as means ± one standard deviation and frequency (n and %) for continuous and categorical variables, respectively. Chi-square and *t*-tests were used for the between-group comparisons of the categorical and continuous variables, respectively.

Time to recurrence and event-free survival curves were analyzed using the Kaplan–Meier method. Cox proportional hazards modeling was used to analyze arrhythmia-free survival with a 3-month blanking period with results presented as hazard ratios (HR) with 95% confidence intervals (CI), after verifying proportionality assumptions. Patients who were lost to follow-up were censored at the date of last known follow-up. Variables in the adjusted model were chosen a priori based on potential for confounding, including age, gender, body mass index (BMI), cardiomyopathy, coronary artery disease, stroke/transient ischemic attack, obstructive sleep apnea, persistent AF, alcohol use, presence of PV reconnections, anti-arrhythmic drug (AAD) use (during the blanking period), any empiric line ablation and any inducible non-PV organized atrial arrhythmia (PAC, AT or macro-reentrant AFL). Two-sided *P* values < 0.05 were considered statistically significant. All analyses were performed using IBM SPSS Statistics Version 28 (IBM Corp, NY, USA).

## Results

**Fig. 1:**
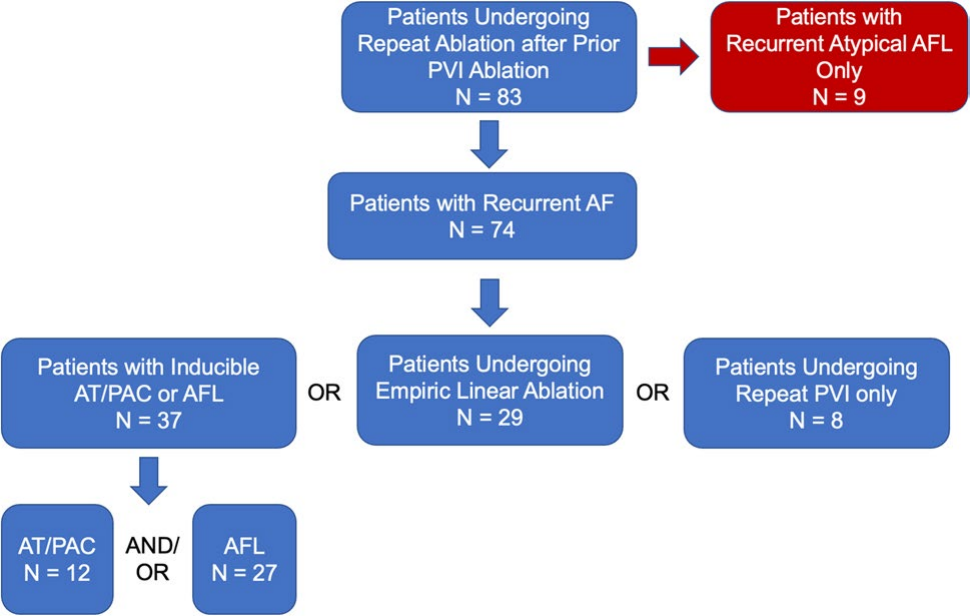
Study Patient Flow

A total of 83 consecutive patients were enrolled into the study. A total of 9 patients were excluded due to only having clinical AFL but not AF. The study patient flow is illustrated in Fig. 1. Table 1 summarizes the clinical and characteristics and echocardiogram data of the study population. The mean age was 66 ± 9 years, with 25% of patients having a history of heart failure, and 59% of patients had persistent AF. Mean left atrial volume index 38 ± 10 ml/m^2^. AAD were used in 41 patients (55%) during the blanking period, and there were no significant differences in AAD use between patients with or without clinical AF recurrence (57% vs 55%, *P* = 0.44). In patients with inducible non-PV organized atrial arrhythmias, 8% were on Class I AAD (flecainide/propafenone), 35% were on Class III AAD (sotalol/dofetilide), and 24% were on amiodarone. In patients without inducible non-PV organized atrial arrhythmias, 8% were on Class I AAD (flecainide/propafenone), 19% were on Class III AAD (sotalol/dofetilide), and 16% were on amiodarone. There were no significant differences in clinical and echocardiographic characteristics between the patients with and without any inducible organized atrial arrhythmia. The characteristics of prior ablation lesions are listed in Table S1 (online supplement).

**Table 1:** Clinical and echocardiographic characteristics of patients

	All patients (*n* = 74)	Any inducible organized atrial arrhythmias (*n* = 37)	No inducible organized atrial arrhythmias (*n* = 37)	*P* value
Age (years)	66 (57–75)	67 (58–76)	65 (57–73)	0.27
Female	20 (27%)	9 (24%)	11 (30%)	0.60
Body mass index (kg/m2)	30 (24–36)	29 (24–34)	31 (25–38)	0.21
Heart failure	19 (25%)	10 (27%)	9 (24%)	0.79
Coronary artery disease	18 (24%)	10 (27%)	8 (22%)	0.59
Cerebrovascular accident/transient ischemic attack	10 (13%)	4 (11%)	6 (16%)	0.50
Obstructive sleep apnea	27 (37%)	14 (38%)	13 (35%)	0.81
Diabetes mellitus	15 (20%)	7 (19%)	8 (22%)	0.77
Most recent creatinine	1.06 (0.71–1.41)	1.02 (0.66–1.38)	1.10 (0.75–1.45)	0.29
Smoking	31 (42%)	17 (46%)	14 (38%)	0.81
Alcohol abuse	20 (27%)	9 (24%)	11 (30%)	0.60
Drug abuse	3 (4%)	2 (5%)	1 (3%)	0.56
Persistent AF	44 (59%)	20 (54%)	24 (65%)	0.16
Paroxysmal AF	30 (41%)	17 (46%)	13 (35%)	*P* = 0.16
LAVI (mL/m2)	38 (28–48)	37 (27–47)	39 (29–49)	*P* = 0.62
LA diameter (cm)	4.3 (3.5–5.1)	4.2 (3.3–5.1)	4.3 (3.6–5.0)	*P* = 0.82
LVEF (%)	60 (48–72)	60 (48–72)	59 (46–72)	*P* = 0.66
LVIDd (cm)	4.9 (4.2–5.6)	5.0 (4.2–5.8)	4.8 (4.2–5.4)	*P* = 0.34
RV failure	9 (16%)	6 (21%)	3 (11%)	*P* = 0.28

Abbreviations: *AF* atrial fibrillation, *LAVI* left atrial volume index, *LA* left atrial, *LVEF* left ventricular ejection fraction, *LVIDd* left ventricular internal diameter in diastole, *RV* right ventricle

### Durability of prior pulmonary vein isolation

In 74 patients with recurrent AF undergoing repeat ablation, PV reconnections were found and ablated in 46 patients (62%). PV reconnections occurred 30% in the right upper PV, 30% in the right lower PV, 23% in the left upper PV, and 18% in the left lower PV. There were 28 patients (38%) who had persistently isolated PVs.

### Prevalence of macro-reentrant atrial flutter

In addition to recurrent AF, macro-reentrant AFLs were identified in 27 patients (36%). As shown in Table 2, a total of 8 patients (29.6%) had CTI-dependent AFL, 11 patients (40.7%) had roof flutters, and the remaining 11 patients (40.7%) had mitral annular flutters. There was no difference in the prevalence of macro-reentrant AFL between patients with and without persistently isolated PVs (Table S2, online supplement).

**Table 2:** Inducible non-PV organized atrial arrhythmias found during repeat AF ablation

	All patients with recurrent AF (*n* = 74)
PV reconnections	46 (62%)
Focal AT/PAC	12 (16%)
Right Atrial AT	1 (6%)
Left Atrial AT	3 (19%)
Right Atrial PAC	5 (31%)
Left Atrial PAC	5 (31%)
Other PAC	2 (17%)
Macro-reentrant atrial flutter	27 (36%)
CTI	8 (27%)
Roof	11 (37%)
Mitral annular	11 (37%)
No inducible Non-PV AT/AFL/PAC	37 (50%)
No inducible Non-PV AT/AFL/PAC or PV Reconnections	15 (20%)

Abbreviations: *PV* pulmonary vein, *AT* atrial tachycardia, *PAC* premature atrial contraction, *CTI* cavo-tricuspid isthmus

### Prevalence of non-pulmonary vein PACs and focal AT

**Fig. 2:**
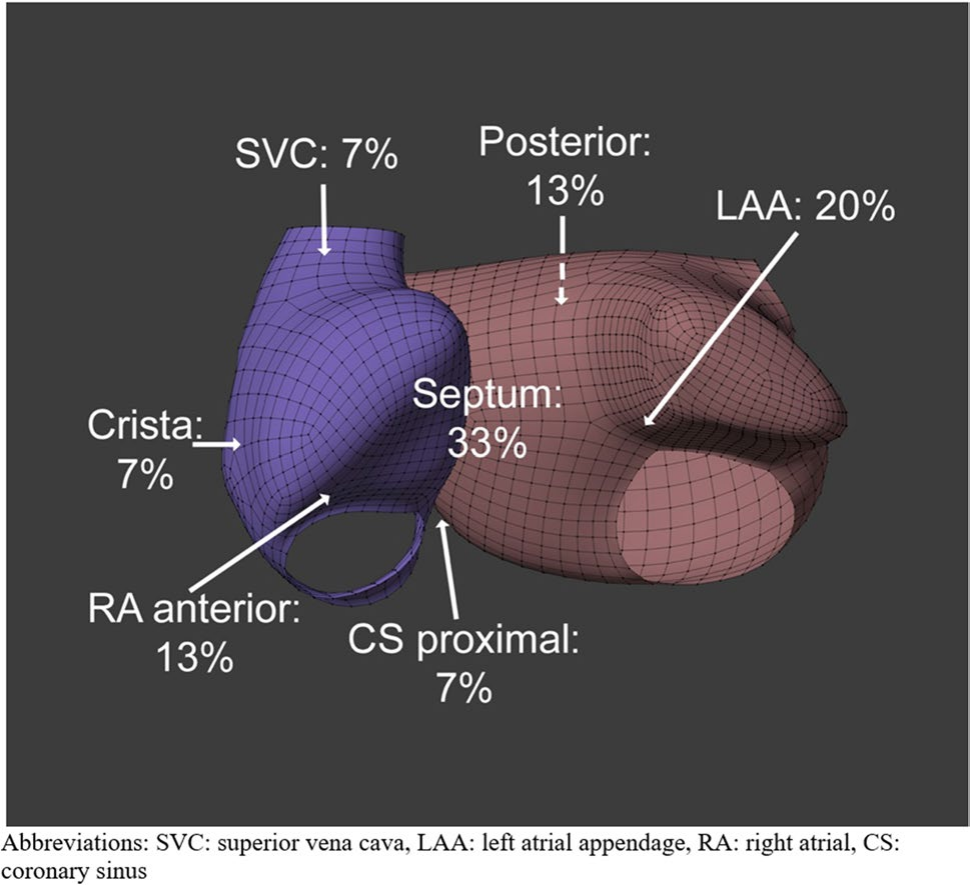
Locations and Prevalence of Non-Pulmonary Vein Premature Atrial Contractions and Atrial Tachycardias

Abbreviations: SVC: superior vena cava, LAA: left atrial appendage, RA: right atrial, CS: coronary sinus

A total of 16 PAC or focal AT sources were identified during sinus rhythm in 12 (15.8%) patients. The locations and distribution of PACs and AT throughout the atria are shown in Fig. 2. Out of the 12 PACs, 5 (41.7%) were located in the left atrium, 5 (41.7%) were located in the right atrium, and 2 (16.6%) were located in other locations (superior vena cava and proximal coronary sinus). Out of the 4 AT, 3 were located in the left atrium and 1 was located in the right atrium. There was no difference in the prevalence of non-PV PAC and focal atrial tachycardias between patients with and without persistently isolated PVs (Table S2, online supplement).

### Clinical outcomes after ablation

**Fig. 3:**
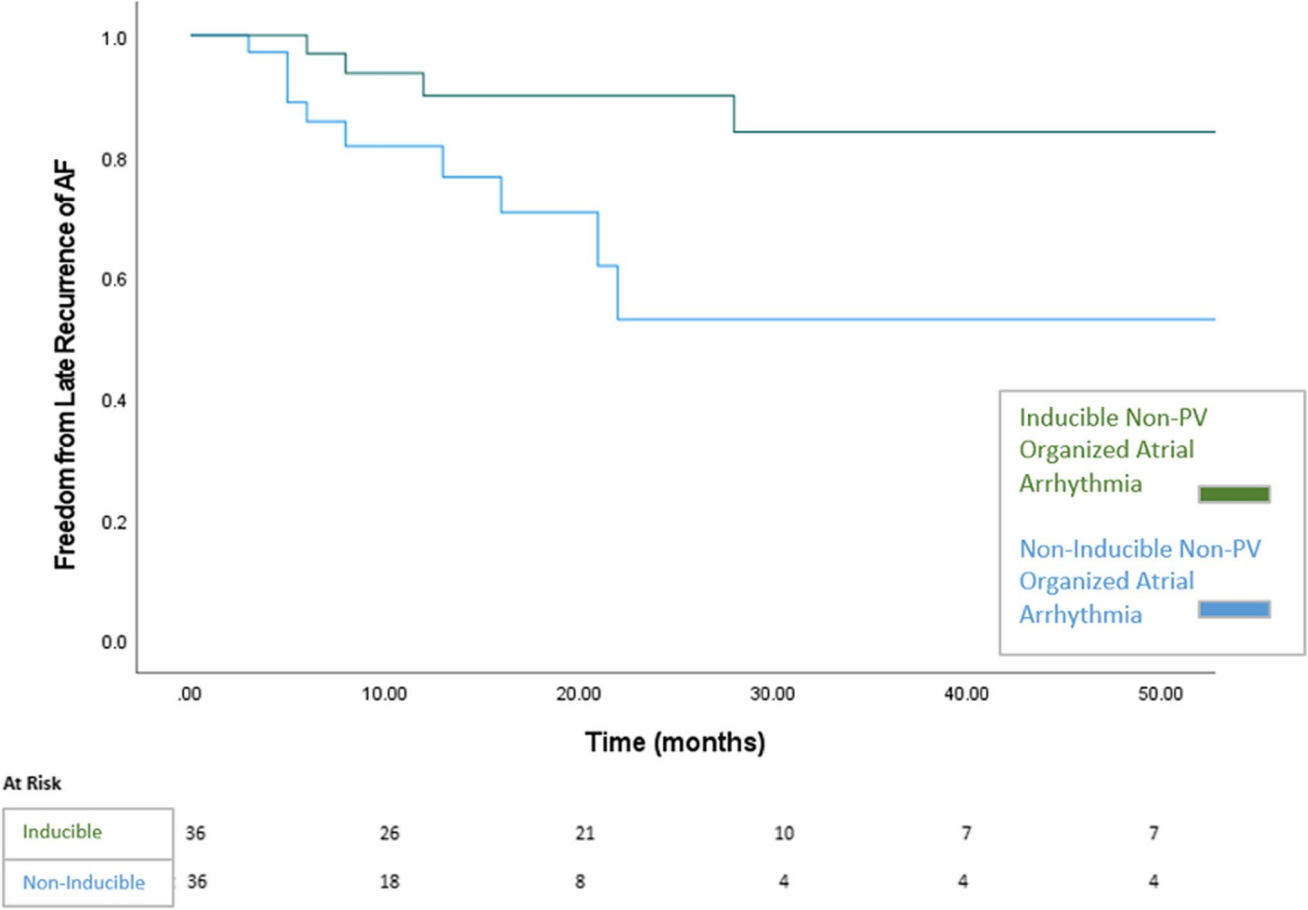
Freedom from Recurrent Clinical AF in Patients With versus Without Inducible Non-PV Organized Atrial Arrythmias (AFL/AT/PAC)

**Fig. 4:**
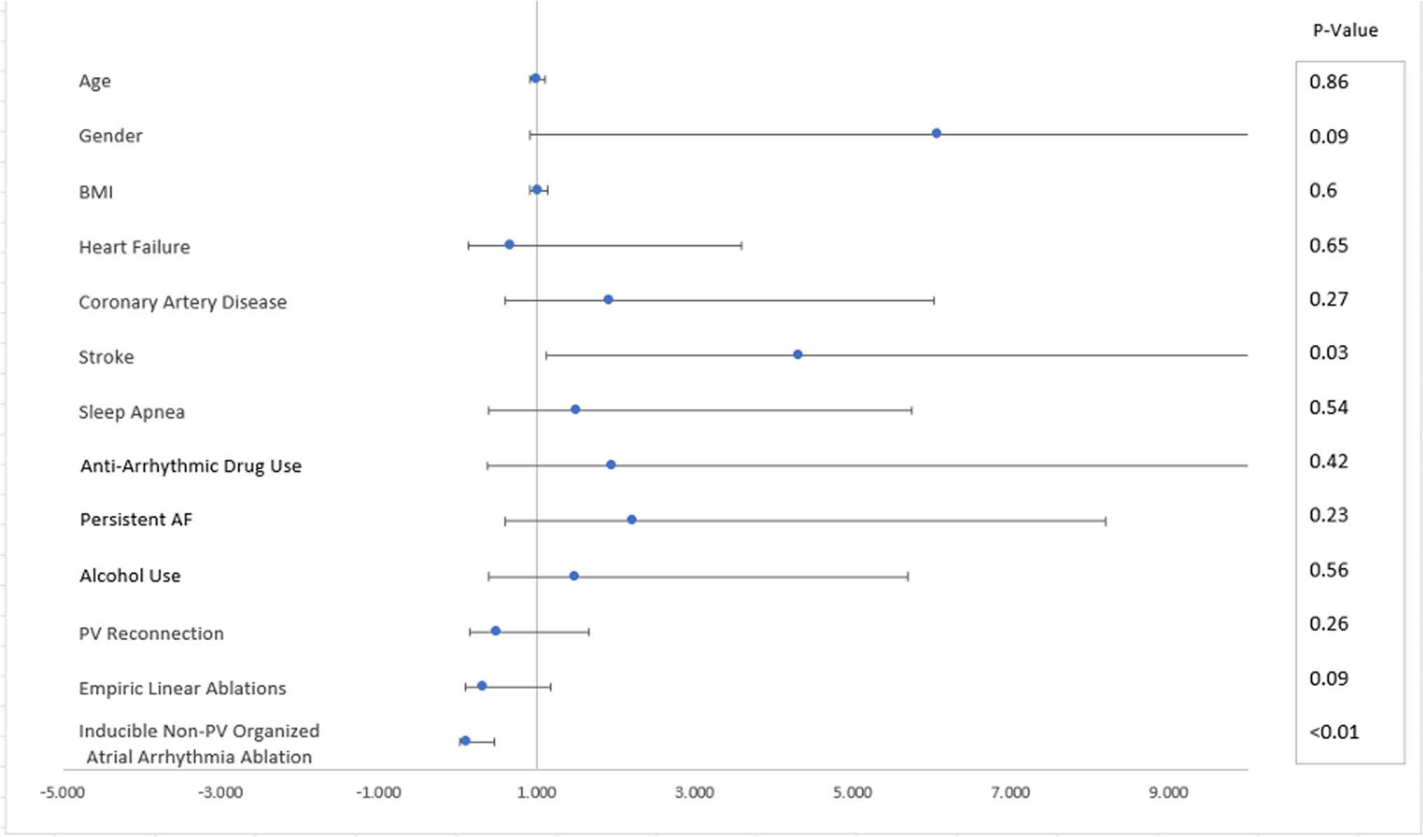
Predictors of Late Clinical AF Recurrence

A total of 55 (74%) patients remained clinical AF free during long-term follow-up of 19 months (interquartile range 9–75 months) as demonstrated by the Kaplan-Meir analysis in Fig. 3 (log-rank < 0.001). Furthermore, 49 (66%) patients did not experience the composite outcome during long-term follow-up. As shown in Table 3, in unadjusted analysis, ablation of mappable non-PV organized atrial arrhythmias was associated with a significant reduction in the recurrence of late AF as compared to no non-PV organized atrial arrhythmias (HR 0.26, 95% CI 0.08–0.85, *P* = 0.03). The association strengthened after multivariable adjustment (HR 0.12, 95% CI 0.03–0.47, *P* < 0.001) as shown in Fig. 4. There was a decreased risk in the composite outcome (late AF, HF hospitalization, and mortality) in those with non-PV organized atrial arrhythmia as compared to no non-PV organized atrial arrhythmia (HR 0.38, 95% CI 0.17–0.85, *P* = 0.02) 0.08–0.85, *P* value 0.03). Findings were comparable after multivariable adjustment (HR 0.29, CI 0.12–0.69, *P* < 0.001).

**Table 3:** Cox-Proportional Hazards Regression Analysis Associating Clinical Outcomes with the Ablation of Inducible Non-PV Organized Atrial Arrhythmias

Outcome	Unadjusted HR (95% Confidence Interval)	P-Value	Adjusted HR (95% Confidence Interval)	P-Value
Recurrence of Late AF	0.26 (0.08–0.85)	0.03	0.12 (0.03–0.47)	< 0.001
Heart Failure Hospitalization	0.01 (0- > 100)	0.61	1.00 (0.01–82.96)	1
Mortality	0.68 (0.13–3.43)	0.64	3.05 (0- > 100)	0.96
Composite Outcome	0.38 (0.17–0.85)	0.02	0.29 (0.12–0.69)	< 0.001

The presence of PV reconnections or empiric linear line ablation was not associated with reductions in clinical AF recurrence (adjusted HR 0.50, CI 0.15–0.1.67, *P* = 0.26 and adjusted HR 0.33, CI 0.09–1.18, *P* = 0.09) or the composite outcome (adjusted HR 0.74, CI 0.26–2.10, *P* = 0.57 and adjusted HR 0.45, CI 0.16–1.25, p = 0.13), as shown in Tables S3-S4 and Figures S1-S2 (online supplement). Furthermore, in Cox regression analysis, AAD use in the blanking period was not associated with decreased clinical AF recurrence (*P* = 0.42). In patients with persistently isolated PVs and inducible non-PV organized atrial arrhythmias, 82% were free from AF.

## Discussion

We demonstrate several findings that advance our understanding of those with recurrent clinical AF undergoing repeat ablation. First, PV reconnections were only found in about half of the patients in our study, and the presence of a PV reconnection as a target for ablation was not associated with reduced freedom from clinical AF. Second, non-PV organized atrial arrhythmias were only found in half of patients with prior durable PV isolation, making this a particularly challenging sub-population to treat. Lastly, mapping and ablation of inducible non-PV organized atrial arrhythmias was associated reduced risk of both late clinical AF and composite outcome of late AF, mortality, and HF hospitalizations.

### PV reconnections

It was previously thought that PV reconnections were the predominant mechanism of recurrent AF in those with prior ablation.^3^ With improvements in ablation technologies, such as contact force and irrigated catheters to deliver durable ablation lesions, more recent studies have shown that the incidence of PV reconnections in recurrent AF may not be as common.^5^ Our data supports this finding as PV reconnections were found only in 61% of patients in our study. This is lower than previous studies that have noted an incidence of PV reconnection in repeat catheter ablations of 75%^11^ to 95% .^3,4^ Interestingly, the presence of PV reconnection was not associated with any reduction in the recurrence of late AF or composite outcome. This may suggest that repeat PV isolation alone may not be a sufficient strategy to prevent the recurrence AF in patients failing prior PV isolation.

In patients who did not have PV reconnections (39%), organized atrial arrhythmias were found in almost half (46%). This is consistent with results from a prior study in which 11 of 27 (41%) of patients with recurrent AF and durable PVI had a non-PV trigger identified.^4^ In our patients with durable PVI and any inducible organized atrial arrhythmias to target for ablation, the majority of these patients (82%) were free from recurrent clinical AF. This data suggests that the presence of inducible targets for ablation contributes to the ability to achieve reasonable freedom from clinical AF in this challenging population.

### Ablation of inducible organized atrial arrhythmias

In contrast, the presence and ablation of any organized atrial arrhythmias was associated with a reduction in AF recurrence and composite endpoint. This supports the hypothesis that any atrial arrhythmia may exacerbate atrial structural and cellular remodeling that further perpetuates the maintenance of AF^12,13,14^. The findings suggest that identification and ablation of all organized atrial arrhythmias may be important in reducing AF recurrence, regardless of whether they directly induce AF. However prospective randomized studies are needed to establish whether non-PV arrhythmia mapping and ablation and use of high dose isoproterenol reduce AF recurrence, and this strategy is currently listed only as a Class IIb indication in the 2017 AF ablation guidelines.^2^

### Empiric linear line ablation

In this study, performance of empiric linear ablation was not associated with reduced AF recurrence or composite endpoint. This is consistent with the results of the STAR-AF2 trial^7^ which showed that empiric linear ablation or CFAE ablation was not associated with decreased AF recurrence. The findings from this study support the importance of identifying electrical mechanisms that may contribute to recurrent AF.

### Clinical AF recurrence in patients with persistently isolated pulmonary veins

There were no identifiable ablation targets for the recurrence of AF in 55% of the patients who did not have PV reconnections. The mechanisms and optimal repeat ablation strategy for these patients are still unknown. Electrically active drivers has been suggested as a possible mechanism maintaining AF.^12^ Focal and rotational driver mapping in patients who had prior PVI resulted in freedom from AF of around 70–80%.^15,16^ However, driver-based ablation is not yet incorporated as a standard protocol in repeat ablations, and further research is needed in this area.^2^ As expected, both inducible and non-inducible organized atrial arrhythmia groups had a high prevalence of the commonly recognized risk factors for AF, including advanced age, obesity, OSA, diabetes, and alcohol use.

## Limitations

First, as a retrospective observational study, causal inferences cannot be made. Second, racial and ethnic description of the population was omitted due to high rate of missingness and unreliability. Third, the modest sample size may be underpowered to detect the effect of different ablation strategies and characteristics on outcomes. Fourth, residual confounding cannot be excluded as multivariable models were adjusted for available risk factors and clinical characteristics. While other factors may play a role in predicting outcomes, such as AAD use following the blanking period^17^, these observations still inform the association between presence and absence of inducible organized atrial arrhythmias in a high-risk cohort. Furthermore, the model assumed fixed covariates and thus does not account for the possibility of time-dependent changes in the covariates for each patient. However, most of the covariates used in this model would not be expected to change over time (i.e., gender, presence of PAC/AT/AFL, presence of PV reconnections). Fifth, all patients received at least a 14-day event monitor (or implantable device interrogation) at 3 months and at 1 year and with any clinical symptoms according to clinical practice at these two institutions. Unfortunately, due to the retrospective study design, the type of post-ablation arrhythmia monitoring after 1 year varied in the cohort and was left to provider discretion. As a result, the recurrence of AF may have been underestimated due to the varying monitoring strategies.

## Conclusions

In patients with recurrent AF after prior ablation, the ablation of inducible non-pulmonary vein organized atrial arrhythmias was associated with the reduction of recurrent late clinical AF in a median follow-up of 1.6 years. Prospective randomized studies are needed to determine the influence of non-PV mechanisms on long-term outcomes in patients with recurrent AF after failed prior AF ablation.

## Disclosures

Dr. Ho: grants from NIH (KL2TR001444), AHA (19CDA34760021), equity in Vektor Medical Inc.

Dr. Han: research support from Abbott.

Dr. Hsu: honoraria from Medtronic, Boston Scientific, Abbott, Biotronik, Biosense-Webster, Pfizer, Bristol-Myers Squibb, Janssen Pharmaceuticals, research grants with Biotronik and Biosense-Webster, and equity interest in Acutus Medical and Vektor Medical.

Dr. Hoffmayer: grants from the NIH (F32 HL10472702 and LRP), consulting for Samsung Electronics, Inc. and Vektor Medical Inc.

Dr. Feld: equity and consulting for Acutus Medical, Adagio, Medwaves, Varian, and co-founded and consulted for Perminova, received CCEP Fellowship Training program stipend support from Medtronic, Biotronik, Biosense Webster, Boston Scientific, and Abbott.

Dr Krummen: grant from the UCSD Galvanizing Engineering in Medicine Foundation and equity in Vektor Medical Inc.

## Supplementary Information

Below is the link to the electronic supplementary material.

Supplementary file1 (DOCX 57 KB)

## Non-standard abbreviations

AF: Atrial fibrillation
PV: Pulmonary veins
PVI: Pulmonary vein isolation
CFAE: Complex fractionated electrograms
AFL: Atrial flutter
AT: Atrial tachycardia
PAC: Premature atrial contractions
BMI: Body mass index
AAD: Anti-arrhythmic drug
