# Supplementary material for: Characteristics and outcomes of recurrent atrial fibrillation after prior failed pulmonary vein isolation
Source: J Interv Card Electrophysiol. 2022 Feb 16;64(3):715–22. doi: 10.1007/s10840-022-01160-w (PMC9378768; doi:10.1007/s10840-022-01160-w)

**Online Supplemental Appendix**

Sai Vanam, MD^1^

Douglas Darden, MD^1^

Muhammad Bilal Munir, MD^1^

Omar Aldaas, MD^1^

Jonathan C. Hsu, MD, MAS, FHRS^1^

Frederick T. Han, MD, FHRS^1,2^

Kurt S. Hoffmayer, MD, PharmD, FHRS^1,2^

Farshad Raissi, MD, FHRS^1^

Ulrika Birgersdotter-Green, MD, FHRS^1^

Gregory K. Feld, MD, FHRS^1^

David E. Krummen, MD, FHRS^1,2^

Gordon Ho, MD, FHRS^1,2^

^1^Department of Medicine, Division of Cardiology, Section of Cardiac Electrophysiology, University of California San Diego, La Jolla, CA

^2^ Veterans Affairs San Diego Medical Center, San Diego, CA

Running Title: *Characteristics and Outcomes of Recurrent AF*

Correspondence to:

Gordon Ho, MD, FACC, FHRS

3350 La Jolla Village Drive

Cardiology Section 111A

San Diego, CA 92161

Email: goho@health.ucsd.edu

Office: 858-246-2972

**Supplemental Index: Table of Contents**

I. Table S1: Characteristics of Prior Ablation Lesions

II. Table S2: Concurrent Atrial Arrhythmias Induced During Repeat AF Ablation

III. Table S3: Cox Regression Analysis of the Recurrence of Late Clinical AF

Table S4: Cox Regression Analysis of the Recurrence of Composite Outcomes

IV. Figure S1: Freedom from Recurrence of Late Clinical AF

Figure S2: Freedom from Recurrence of Late AF, HF Hospitalizations and Death

I. Table S1: Characteristics of Prior Ablation Lesions

|  | **All patients (n=74)** |
| --- | --- |
| Prior WACA | 69 (93%) |
| Prior Segmental | 17 (23%) |
| CTI | 52 (70%) |
| Any LA linear ablation (not CTI) | 32 (44%) |
| Anterior Mitral Isthmus Line | 3 (4%) |
| Posterior Mitral Isthmus Line | 7 (10%) |
| Roof line | 23 (32%) |
| Posterior wall isolation (Roof and Floor line) | 2 (3%) |
| Other (CFAE, PAC, etc) | 9 (12%) |

II. Table S2: Concurrent Atrial Arrhythmias Induced During Repeat AF Ablation

|  | **All patients (n=74)** | **PV Reconnections (n=46)** | **No PV Reconnections (n=28)** | **P- Value** |
| --- | --- | --- | --- | --- |
| PV reconnections | 46 (62%) | 46 (100%) | 0% | P = <0.01 |
| Focal Mechanism | 12 (16%) | 9 (20%) | 3 (11%) | P = 0.23 |
| RA Atrial Tachycardia | 1 (6%) | 0 | 1 (20%) | P = 0.72 |
| LA Atrial Tachycardia | 3 (19%) | 2 (18%) | 1 (20%) | P = 0.87 |
| RA Premature Atrial Contraction | 5 (31%) | 4 (36%) | 1 (20%) | P = 0.17 |
| LA Premature Atrial Contraction | 4 (25%) | 2 (18%) | 2 (40%) | P = 0.72 |
| Other | 3 (19%) | 3 (27%) | 0 | P = 0.59 |
| Macro-reentrant Atrial Flutter | 27 (36%) | 18 (39%) | 9 (31%) | P = 0.55 |
| CTI | 8 (27%) | 6 (30%) | 2 (15%) | P = 0.43 |
| Roof | 11 (37%) | 6 (30%) | 5 (38%) | P = 0.57 |
| Mitral Annular | 11 (37%) | 8 (40%) | 6 (46%) | P = 0.67 |

III. Table S3: Cox Regression Analysis of the Recurrence of Late Clinical AF

| **Recurrence of Late AF** |  |  |  |  |
| --- | --- | --- | --- | --- |
|  | Unadjusted HR | P-Value | Adjusted HR | P-Value |
| Age | 1.01 (0.95-1.08) | 0.78 | 1.00 (0.92-1.11) | 0.86 |
| Gender | 4.29 (0.56-33.09) | 0.16 | 6.08 (0.77-48.20) | 0.09 |
| BMI | 1.03 (0.94-1.12) | 0.55 | 1.02 (0.92-1.14) | 0.68 |
| Heart Failure | 0.74 (0.16-3.32) | 0.69 | 0.68 (0.13-3.59) | 0.65 |
| Coronary Artery Disease | 1.99 (0.67-5.93) | 0.22 | 1.92 (0.61-6.03) | 0.27 |
| Stroke | 2.56 (0.71-9.31) | 0.15 | 4.32 (1.12-16.62) | 0.03 |
| Sleep Apnea | 1.63 (0.57-4.65) | 0.36 | 1.51 (0.40-5.75) | 0.54 |
| Persistent AF | 1.49 (0.47-4.75) | 0.51 | 2.22 (0.60-8.20) | 0.23 |
| Alcohol | 2.39 (0.83-6.92) | 0.11 | 1.49 (0.39-5.70) | 0.56 |
| PV Reconnection | 0.61 (0.21-1.73) | 0.35 | 0.50 (0.15-1.67) | 0.26 |
| Any Empiric Linear Ablation | 0.77 (0.24-2.45) | 0.65 | 0.33 (0.09-1.18) | 0.09 |
| Any Inducible Organized Atrial Arrhythmia Ablation | 0.26 (0.08-0.85) | 0.03 | 0.12 (0.03-0.47) | <0.01 |

Table S4: Cox Regression Analysis of the Composite Outcome

| **Composite outcome** |  |  |  |  |
| --- | --- | --- | --- | --- |
|  | Unadjusted HR | P-Value | Adjusted HR | P-Value |
| Age | 1.04 (0.99-1.10) | 0.15 | 1.02 (0.95-1.11) | 0.52 |
| Gender | 1.53 (0.52-4.55) | 0.44 | 1.48 (0.45-4.83) | 0.52 |
| BMI | 1.00 (0.93-1.07) | 0.94 | 1.02 (0.95-1.10) | 0.56 |
| Heart Failure | 1.72 (0.67-4.46) | 0.26 | 1.56 (0.56-4.31) | 0.39 |
| Coronary Artery Disease | 1.87 (0.82-4.25) | 0.14 | 2.53 (1.08-5.96) | 0.03 |
| Stroke | 2.67 (0.98-7.23) | 0.05 | 3.16 (1.13-8.84) | 0.03 |
| Sleep Apnea | 0.71 (0.30-1.66) | 0.43 | 0.76 (0.28-2.06) | 0.59 |
| Persistent AF | 1.83 (0.75-4.48) | 0.18 | 1.76 (0.71-4.36) | 0.22 |
| Alcohol | 1.43 (0.61-3.33) | 0.41 | 0.82 (0.30-2.24) | 0.70 |
| PV Reconnection | 0.95 (0.42-2.13) | 0.90 | 0.74 (0.26-2.10) | 0.57 |
| Any Empiric Linear Ablation | 1.17 (0.52-2.67) | 0.70 | 0.45 (0.16-1.25) | 0.13 |
| Any Inducible Organized Atrial Arrhythmia Ablation | 0.38 (0.17-0.85) | 0.02 | 0.29 (0.12-0.69) | <0.01 |

IV. Figure S1: Freedom from Recurrence of Late Clinical AF


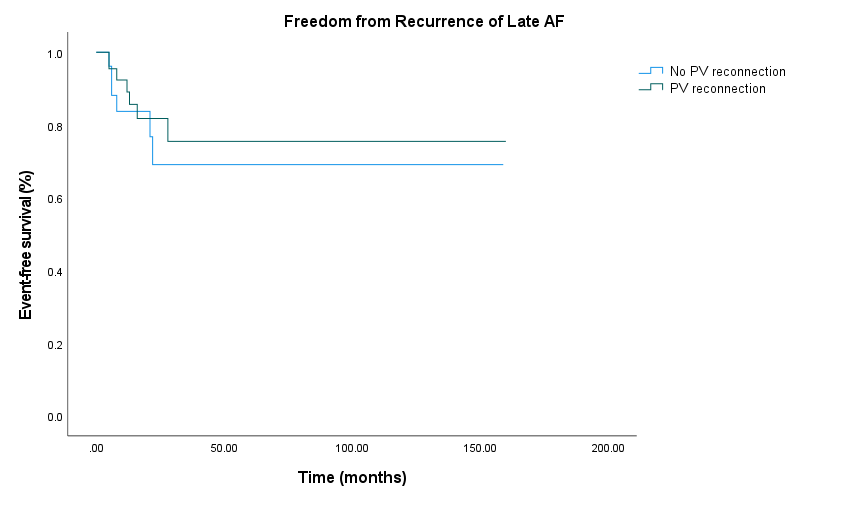


Figure S2: Freedom from Recurrence of Late AF, HF Hospitalizations and Death


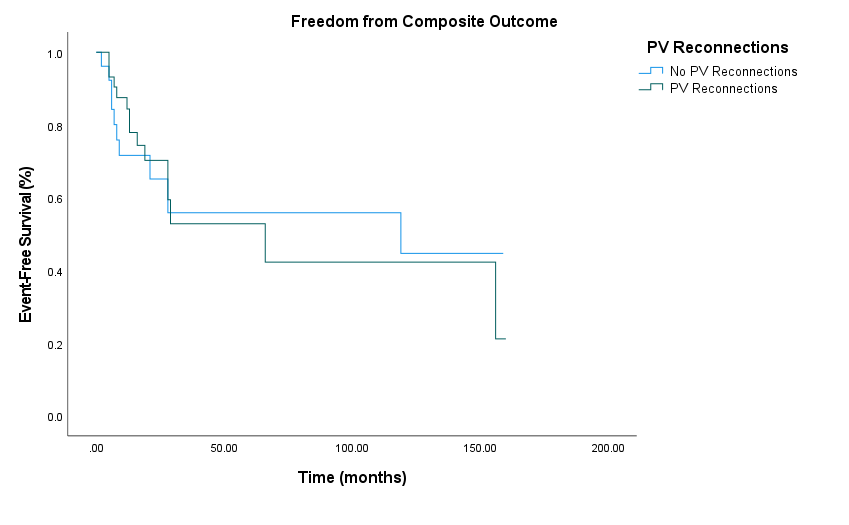

Supplement: Supplementary file 1 — Supplementary file1 (DOCX 57 KB) [file 10840_2022_1160_MOESM1_ESM.docx]
